# Lung Nodule Detection and False Positive Reduction Using Multi-View and Background Images

**DOI:** 10.3390/s26196246

**Published:** 2026-10-01

**Authors:** Leandro Silva, Helio Pedrini

**Affiliations:** 1 Venturus—Innovation & Technology, Campinas 13086-530, Brazil; 2 Institute of Computing, University of Campinas, Campinas 13083-970, Brazil; helio@ic.unicamp.br

**Keywords:** lung nodule detection, deep learning, medical image analysis, false positive reduction, multi-view detection, YOLOv8x

## Abstract

This paper presents a lung nodule detection and false positive reduction approach based on YOLOv8x, multi-view CT representations, and background slices used as negative samples. Lung cancer remains one of the leading causes of cancer-related mortality worldwide, and early detection is essential to improve patient survival. Although computed tomography (CT) provides detailed three-dimensional information about the lungs, the large number of generated slices increases the complexity of radiological analysis and can contribute to diagnostic errors. To address this challenge, Computer-Aided Detection (CAD) systems have been developed to assist radiologists in identifying suspicious regions. In this work, we combine axial, coronal, and sagittal representations in a single YOLOv8x detection pipeline, use additional LIDC-IDRI annotation clusters with fewer than three radiologist markings as auxiliary positive samples, and introduce nodule-free background slices as negative training samples. Detections are consolidated through intra-view clustering and weighted inter-view fusion. Experiments following the LUNA16 cross-validation protocol show that the proposed strategy improves detection performance while substantially reducing false positives. The axial baseline achieved a Competition Performance Metric (CPM) score of 0.553 with 32.61 candidates per scan, whereas the multi-view configuration with a nominal budget of 20,000 background images achieved a CPM score of 0.863 and reduced the average number of candidates to 9.11 per scan while preserving high sensitivity. These results show that the combined use of multiple anatomical views and background-negative training can substantially reduce spurious candidates in CT-based lung nodule detection.

## 1. Introduction

Lung cancer remains one of the most important public health challenges worldwide due to its high incidence and mortality. According to the latest estimates from the World Health Organization (WHO) and the International Agency for Research on Cancer (IARC), approximately 20 million new cancer cases and 9.7 million cancer-related deaths occurred worldwide in 2022, with the global cancer burden projected to exceed 35 million new cases by 2050 [1]. Lung cancer was the most commonly diagnosed cancer and the leading cause of cancer death, accounting for approximately 2.5 million new cases and 1.8 million deaths in 2022 [2]. Because lung cancer is often diagnosed at advanced stages, early detection remains essential to improve patient outcomes. Recent SEER statistics report a 5-year relative survival rate of 29.5% for lung and bronchus cancer overall, increasing to 65.5% for localized disease but decreasing to 10.5% for distant-stage disease, highlighting the importance of accurate and early detection methods [3].

Chest radiography [4,5] has traditionally been used for detecting lung diseases; however, computed tomography (CT), introduced into clinical practice in the 1970s, provides substantially more detailed images. CT generates hundreds of two-dimensional (2D) slices that together represent a three-dimensional (3D) volume of the chest, allowing precise visualization of the lungs. The amount of information contained in a CT examination, however, increases the complexity of radiological analysis and motivates the use of automated assistance.

Computer-Aided Detection (CAD) systems assist radiologists by identifying suspicious regions that should receive closer inspection. A CAD pipeline for lung nodule detection typically includes a sensitive candidate-detection stage followed by mechanisms intended to reduce false positives (FPs). With the development of deep learning, convolutional neural networks (CNNs) [6] and object detectors have substantially improved candidate detection and false-positive reduction.

Previous studies have used multiple anatomical projections or other 2D views to exploit the 3D context of CT data [7,8]. We investigate the combined effect of three elements within a single YOLOv8x-based detector: (i) training and inference using the three orthogonal anatomical views, (ii) explicit training with nodule-free CT slices as background-negative samples, and (iii) score-level fusion of spatially corresponding candidates across views. We further provide ablation experiments on the amount of background data and on the inter-view score weights. Under the LUNA16 evaluation protocol [9], the resulting configuration reaches a CPM of 0.863 while substantially reducing the average number of generated candidates per scan.

## 2. Background

Early lung-nodule CAD systems relied heavily on empirically defined Hounsfield-unit thresholds, hand-crafted feature extraction, segmentation rules, and subsequent region classification. Their performance therefore depended strongly on manually designed representations and domain-specific processing [10,11].

Convolutional Neural Networks (CNNs) [6] reduced the dependence on manually designed features by learning task-specific representations directly from data. Several studies subsequently used CNNs to reduce false positives in lung nodule detection. Setio et al. [7] proposed a multi-view CNN for false-positive reduction using nine symmetrical views of a 3D candidate region. Wang et al. [8] proposed a three-branch multi-view CNN for lung nodule segmentation using axial, coronal, and sagittal representations. These studies demonstrate that complementary 2D projections can preserve useful 3D contextual information while retaining the computational advantages of 2D models.

With the introduction of Fast R-CNN [12] and YOLO [13], object detectors became increasingly relevant to CAD systems. Because CT scans are volumetric, other studies instead adopted explicitly 3D architectures. Dou et al. [14] introduced a two-stage system with a fully convolutional 3D network for sensitive candidate detection. Zhu et al. [15] used a dual-path 3D Faster R-CNN together with a U-Net-like encoder–decoder structure. Xie et al. [16] adapted a 2D Faster R-CNN using two region proposal networks and a deconvolutional layer. Zhang et al. [17] proposed a 3D Faster R-CNN based on multi-scale attention blocks and a U-Net-like encoder–decoder. Liu et al. [18] introduced 3D ARCNN, which uses asymmetric residual structures and multi-layer cascades, whereas Jian et al. [19] presented 3DAGNet with spatial-attention, global-search, and multi-scale contextual modules.

In this work, we study whether a 2D YOLOv8x detector can exploit volumetric context through orthogonal views while simultaneously learning from nodule-free slices as explicit negative examples. The contribution is the combined training and fusion strategy and its ablation under the LUNA16 protocol.

## 3. Lung Nodule Detection Method

**Figure 1:**
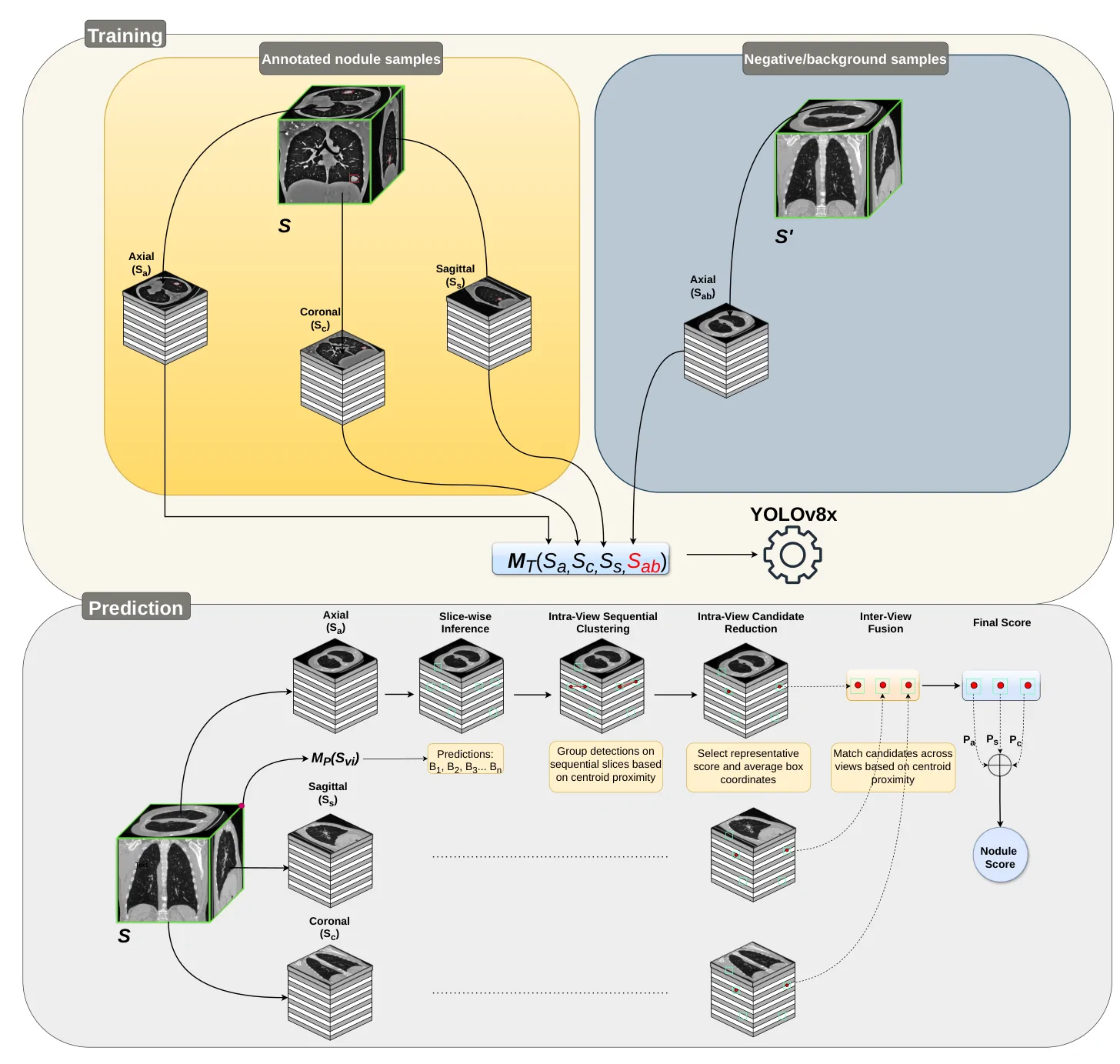
Proposed architecture. The upper section illustrates the Training phase, in which YOLOv8x is fine-tuned using axial, coronal, sagittal, and axial background-negative CT images. The lower section illustrates the Prediction pipeline, including slice-wise inference, intra-view clustering, candidate reduction, inter-view fusion, and final nodule scoring.

The proposed architecture has two main stages, Training and Prediction, as shown in Figure 1. During Training, a YOLOv8x detector is fine-tuned using annotated nodule images from three orthogonal CT views together with nodule-free background slices used as negative samples. During Prediction, the trained detector is applied independently to axial, coronal, and sagittal slices, after which detections are consolidated by intra-view clustering and inter-view fusion.

### 3.1. Dataset and Auxiliary LIDC-IDRI Nodules

This study uses the LUNA16 dataset [9], which is derived from LIDC-IDRI. LIDC-IDRI contains 1018 thoracic CT scans, whereas LUNA16 retains 888 scans after applying its inclusion criteria, including a maximum slice thickness of 2.5 mm. The official LUNA16 positive set contains 1186 pulmonary nodules accepted by at least three radiologists. The dataset is distributed in 10 predefined subsets that are used for cross-validation. Each official LUNA16 nodule annotation provides the CT series UID, the *X*, *Y*, and *Z* coordinates of the nodule center in physical space, and the nodule diameter in millimeters.

The additional positive samples used in this work were not obtained from an independent set of CT scans. For each LUNA16 CT series, identified by its SeriesInstanceUID, the corresponding LIDC-IDRI radiologist annotation clusters were retrieved. Clusters with three or more radiologist annotations correspond to the official LUNA16 positive set, whereas clusters with fewer than three radiologist annotations are excluded from the official LUNA16 positive-nodule set. We retained 1047 of these excluded radiologist annotation clusters as an auxiliary positive set for training.

### 3.2. Data Imbalance

Nodules were grouped by diameter into small (D<5 mm), medium (5≤D<20 mm), and large (D≥20 mm) categories, corresponding to the size strata considered in [17]. The distribution of medium-sized nodules is imbalanced relative to small nodules, with a ratio of approximately 3.2:1. To increase the representation of small nodules, we estimated the diameter *D* of the auxiliary LIDC-IDRI annotation clusters using a tetrahedral volume approximation and the diameter of a sphere with equivalent volume:

$$D={\left(\frac{6V}{\pi }\right)}^{1/3},\;V=\left|\sum_{i=0}^{N}\frac{{\vec{a}}_{i}\cdot \left({\vec{b}}_{i}\times {\vec{c}}_{i}\right)}{6}\right|,$$

where *V* is the estimated nodule volume and ${\vec{a}}_{i}$, ${\vec{b}}_{i}$, and ${\vec{c}}_{i}$ are vectors associated with the vertices of triangles extracted from the nodule surface mesh.

We compared the estimated diameters with the official LUNA16 diameters and obtained the root mean square error (RMSE) values shown in Table 1. The method was then used to categorize the auxiliary LIDC-IDRI clusters (Table 2). Combining the 1186 official LUNA16 nodules (Table 3) with the 1047 auxiliary clusters produced a complete pool of 2233 positive nodule instances before fold-specific training selection and reduced the medium-to-small ratio to approximately 1.72:1 (Table 4).

### 3.3. Object Detection Model

YOLO (You Only Look Once) [13] is a family of single-stage object detectors that jointly predict object locations and class probabilities. The experiments reported in this work use the YOLOv8x detector from Ultralytics [20]. Ultralytics training also supports images with no object annotations; such images act as background-negative examples. We use this mechanism with CT slices that do not contain an official LUNA16 nodule in the sampled position, allowing the detector to learn from nodule-free anatomical structures that might otherwise produce spurious candidates.

The YOLOv8 detection loss includes bounding-box regression, Distribution Focal Loss, and classification components:

$$\mathrm{Loss}={\mathrm{Loss}}_{\mathrm{box}}+{\mathrm{Loss}}_{\mathrm{dfl}}+{\mathrm{Loss}}_{\mathrm{cls}}.$$

### 3.4. CT Pre-Processing, Background Sampling, and Training

CT volumes were loaded with SimpleITK while preserving their original voxel spacing and origin. The preprocessing path handled two intensity encodings observed in the data. If more than half of the voxels in a volume were positive, negative values were set to zero. Otherwise, values below −1024 were clipped to −1024 and the volume was shifted by +1024. Each complete volume was then min–max normalized to the range [0,255] and converted to uint8. No fixed lung-window interval was applied. The training volumes were not resampled to isotropic spacing; the original spacing and origin were retained for physical-coordinate calculations. Each grayscale 2D slice was replicated into three channels before being provided to YOLOv8x.

Positive training images were generated from the axial, coronal, and sagittal planes at the corresponding nodule location. Background-negative slices were generated from the same LUNA16 CT population. For scans containing official LUNA16 nodules, slice positions intersecting the slice extent of an official nodule annotation were excluded from background generation. Candidate background positions were sampled across each volume at regularly spaced relative offsets, and nodule-free scans were sampled with the same procedure. For the final experiments reported here, only axial background slices were used for training.

Background selection was performed independently within each cross-validation iteration. Only background images belonging to the nine training subsets were eligible; the test subset was excluded. The requested background budget was distributed approximately uniformly across the nine training subsets. The ablation experiments considered nominal budgets of 5k, 10k, 15k, and 20k background images.

All experiments used YOLOv8x initialized from Ultralytics pre-trained weights and four NVIDIA GeForce RTX 3070 Ti GPUs. The training hyperparameters were kept fixed across the cross-validation folds: lr0 = 0.0001, lrf = 0.1, momentum = 0.937, weight_decay = 0.0005, the Adam optimizer, 30 epochs, and a total batch size of 48 distributed over the four GPUs. Data augmentation used hsv_h = 0.015, hsv_s = 0.7, hsv_v = 0.4, degrees = 0.2, translate = 0.2, scale = 0.2, shear = 0.1, flipud = 0.3, fliplr = 0.5, and mosaic = 1.0. Deterministic training was enabled.

Ten-fold cross-validation was performed using the ten predefined LUNA16 subsets. For each fold, nine LUNA16 subsets were used for training and the remaining subset was used for evaluation. This partitioning was applied to the official LUNA16 positive samples and to the background samples derived from those subsets. The 1047 auxiliary LIDC-IDRI positive clusters were handled separately: the same auxiliary set was added to the training data in every fold and was not partitioned according to the LUNA16 subset structure.

## 4. Prediction

Let $S\in R^{H\times W\times D}$ denote a preprocessed CT volume and let V={a,c,s} denote the axial, coronal, and sagittal views. For each view v∈V, the volume is represented as an ordered sequence of 2D slices,

(1) $$S_{v}={\left\{s_{v,i}\right\}}_{i=1}^{N_{v}}.$$

The trained YOLOv8x prediction model $M_{P}$ is applied independently to every slice,

(2) $$M_{P}\left(s_{v,i}\right)\to B_{v,i},$$

where $B_{v,i}$ is the set of 2D detections produced for slice *i* of view *v*. Each detection contains a bounding box, a confidence score, and a centroid. The 2D centroid together with the slice index is mapped to the original CT physical-coordinate system using the scan spacing and origin, so detections from all three views can be compared in a common 3D coordinate system.

Before cross-slice grouping, non-maximum suppression is applied independently to each slice with an intersection-over-union threshold of 0.1 and a maximum of 100 retained boxes. The detections from a complete view are then collected as

(3) $$B_{v}=\overset{N_{v}}{\underset{i=1}{⋃}}B_{v,i}.$$

Within each view, detections are ordered by slice position and grouped into sequential chains according to the proximity of their physical 3D centroids. For centroids $c_{i}=\left(x_{i},y_{i},z_{i}\right)$ and $c_{j}=\left(x_{j},y_{j},z_{j}\right)$, the implementation evaluates the squared Euclidean distance

(4) $$d^{2}\left(c_{i},c_{j}\right)={\left(x_{i}-x_{j}\right)}^{2}+{\left(y_{i}-y_{j}\right)}^{2}+{\left(z_{i}-z_{j}\right)}^{2},$$

and associates detections when

(5) $$d^{2}\left(c_{i},c_{j}\right)<\tau ,\;\tau =30\;{\mathrm{mm}}^{2}.$$

Thus, the numerical criterion used by the implementation is equivalent to a Euclidean centroid distance smaller than $\sqrt{30}\approx 5.48$ mm. This threshold was fixed throughout all cross-validation folds and reported experiments.

Let $C_{k}^{v}$ denote the *k*th intra-view chain in view *v*. Each chain is reduced to one 3D candidate $N_{k}^{v}$. The highest-confidence detection supplies the representative confidence score, while the bounding-box coordinates are averaged over the detections in the chain to obtain the candidate localization. This produces one candidate set for each view,

(6) $$N_{v}=\left\{N_{1}^{v},\ldots ,N_{K_{v}}^{v}\right\},\;v\in V.$$

The final stage is inter-view fusion. Candidate sets from the three views are considered jointly,

(7) $$N_{\mathrm{all}}=N_{a}\cup N_{c}\cup N_{s}.$$

Candidates originating from different views are associated in the same physical-coordinate system using the centroid-proximity rule in Equation (5). The resulting fused groups are denoted by $F=\{F_{1},\ldots ,F_{M}\}$, with each group containing at most one candidate from each anatomical view.

For a fused group $F_{m}$, let $p_{a}$, $p_{c}$, and $p_{s}$ denote the confidence scores of its axial, coronal, and sagittal candidates, respectively, with corresponding weights $w_{a}$, $w_{c}$, and $w_{s}$. The general weighted fusion score is

(8) $$P_{m}=\frac{w_{a}p_{a}+w_{c}p_{c}+w_{s}p_{s}}{w_{a}+w_{c}+w_{s}}.$$

The experiments in Section 5.3 selected the axial:coronal:sagittal weighting 5:1:1, giving

(9) $$P_{m}=\frac{5p_{a}+p_{c}+p_{s}}{7}.$$

If one or more views are not represented in $F_{m}$, the corresponding score is set to zero and the denominator remains 7. Therefore, candidates supported by fewer views are penalized instead of being re-normalized over only the available views.

## 5. Experimental Results

This section reports the experiments performed with the YOLOv8x detector (Ultralytics Inc., Frederick, MD, USA).

### 5.1. Evaluation Metrics

Performance was evaluated with the LUNA16 Free-Response Receiver Operating Characteristic (FROC) protocol, sensitivity, and the Competition Performance Metric (CPM). Sensitivity is defined as

(10) $$\mathrm{Sensitivity}=\frac{\mathrm{TP}}{\mathrm{TP}+\mathrm{FN}}.$$

where TP and FN denote true-positive and false-negative detections, respectively.

The FROC curve plots sensitivity against the average number of false positives per scan. CPM is the mean sensitivity at seven predefined false-positive rates: 0.125, 0.25, 0.5, 1, 2, 4, and 8 FPs/scan. These operating points include the 1–4 FPs/scan region commonly used to interpret practical CAD operating conditions in the LUNA16 evaluation framework [9].

Following the LUNA16 evaluation procedure, FROC uncertainty was estimated by scan-level bootstrapping with 1000 resamples with replacement and a 95% confidence level [9]. For each bootstrap sample, scans—and therefore the candidates belonging to those scans—are resampled with replacement, the FROC curve is recomputed and interpolated on a common false-positive-per-scan axis, and the mean, lower, and upper sensitivity bounds are obtained. In the evaluation implementation, the reported CPM is calculated from the bootstrap-mean sensitivities at the seven standard operating points.

Predictions from the ten LUNA16 test folds were pooled before computing the FROC curve and CPM. Therefore, the reported CPM was calculated from the aggregate cross-validated predictions rather than as the arithmetic mean of ten fold-specific CPM values.

### 5.2. Candidate Detection and Background Ablation

The full positive image pool comprised 2233 axial images for the baseline and 6699 images for the multi-view experiment (2233 per anatomical view). These totals describe the complete dataset before the fold-specific training selection detailed in Section 3.4. The axial baseline achieved a CPM of 0.553, a sensitivity of 0.908, and 32.61 candidates per scan. The three-view model achieved a CPM of 0.702, a sensitivity of 0.933, and 31.09 candidates per scan.

**Figure 2:**
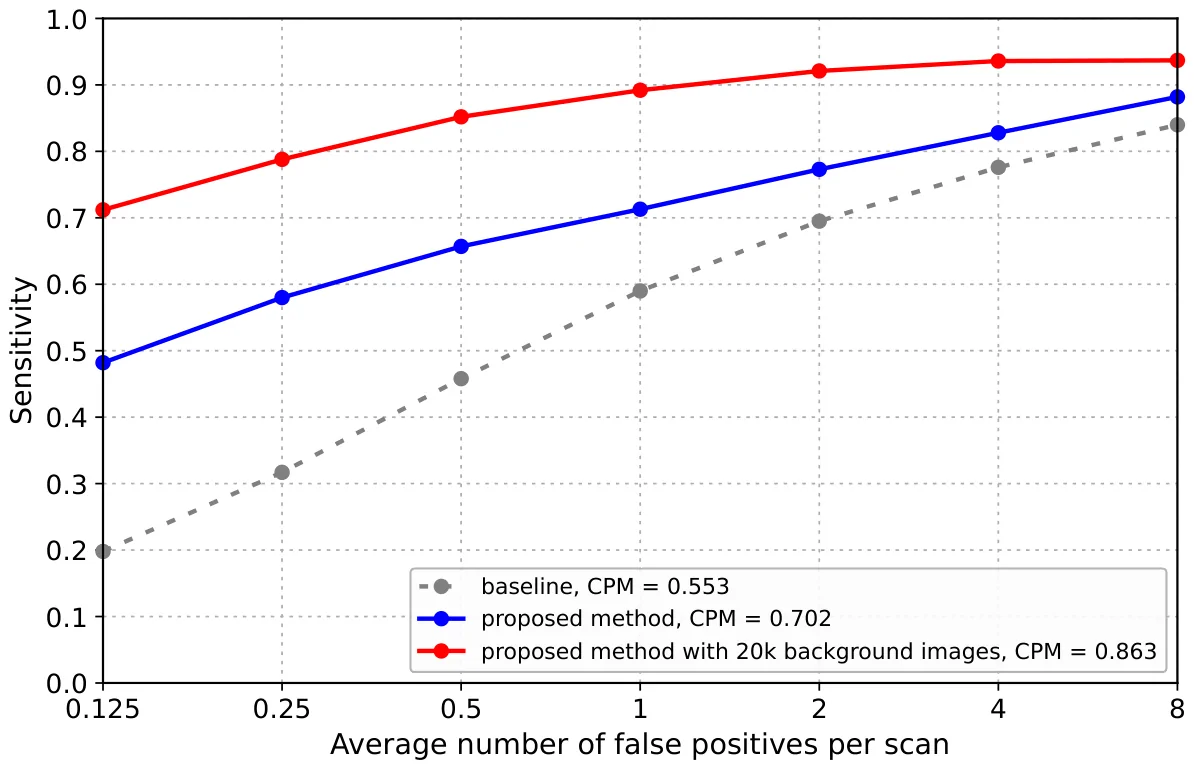
FROC curves for the axial baseline and the proposed multi-view/background configurations on LUNA16.

The increase from 0.553 to 0.702 reflects the combined effect of using three anatomical views and increasing the number of 2D training representations per nodule. The individual contributions of these two factors were not isolated in the current experiments. The FROC curves for the baseline, the proposed method, and the proposed method with 20k background images are shown in Figure 2.

We then evaluated the effect of adding axial background-negative samples while keeping the three-view positive configuration. Table 5 shows a progressive reduction in the average number of generated candidates as the nominal background budget increases from 5k to 20k. CPM increased from 0.702 without background samples to 0.832, 0.844, 0.847, and 0.863 for the 5k, 10k, 15k, and 20k configurations, respectively. Exploratory experiments with background budgets above 20k were also performed, but they did not show a clear additional performance improvement. Because the numerical results of these exploratory runs were not retained, they are not included in the quantitative comparison. Larger background sets also increased training time and computational cost. Therefore, 20k was retained as the largest reported configuration and should be interpreted as a practical operating point rather than as a theoretically optimal background quantity.

The final 20k configuration achieved 9.11 candidates per scan, sensitivity of 0.937, and CPM of 0.863. Relative to the axial baseline, this represents a 56% increase in CPM together with a substantial reduction in candidate burden. Relative to the three-view configuration without background samples, CPM increased by approximately 23%.

### 5.3. Inter-View Weight Selection

The axial contribution to the fused candidate score was evaluated through a grid search in which the coronal and sagittal weights were fixed at 1 and the axial weight was varied from 2 to 6. The spatial association rule was held fixed for all weighting experiments: intra-view clustering and inter-view matching both used the criterion in Equation (5). Only the score weights were changed. For each candidate weighting, the complete 10-fold cross-validation protocol was executed and the aggregate CPM over the ten held-out subsets was compared. The 5:1:1 and 6:1:1 axial:coronal:sagittal weightings produced the highest aggregate CPM to the reported precision (0.863). The 5:1:1 weighting was retained as the operating configuration used in the reported experiments (Table 6).

The weighting was not selected on a separate validation set. Because the same cross-validation results were used both to select the fusion weights and to estimate performance, the reported performance of the selected configuration may be optimistic. We therefore regard 5:1:1 as an empirically selected operating configuration rather than an independently validated optimum.

### 5.4. Comparison with Existing LUNA16 Methods

Table 7 compares the proposed YOLOv8x multi-view method with published approaches that report results using the LUNA16 FROC operating points. Our method achieves a CPM of 0.863, compared with 0.856 for Zhang et al. [17], 0.880 for Jian et al. [19], and 0.912 for Liu et al. [18]. At 1, 2, and 4 FPs/scan, our method achieves sensitivities of 0.892, 0.921, and 0.936, respectively, compared with 0.866, 0.917, and 0.929 reported by Zhang et al. at the same operating points. Although the methods in Table 7 are evaluated using the LUNA16 FROC protocol, they differ in training-data composition, preprocessing, model architecture, augmentation, candidate-generation strategy, use of ensembles or auxiliary data, and training protocol. In particular, our training procedure supplements the official LUNA16 positives with auxiliary LIDC-IDRI annotation clusters.

### 5.5. Nodule Size and Error Analysis

**Figure 3:**
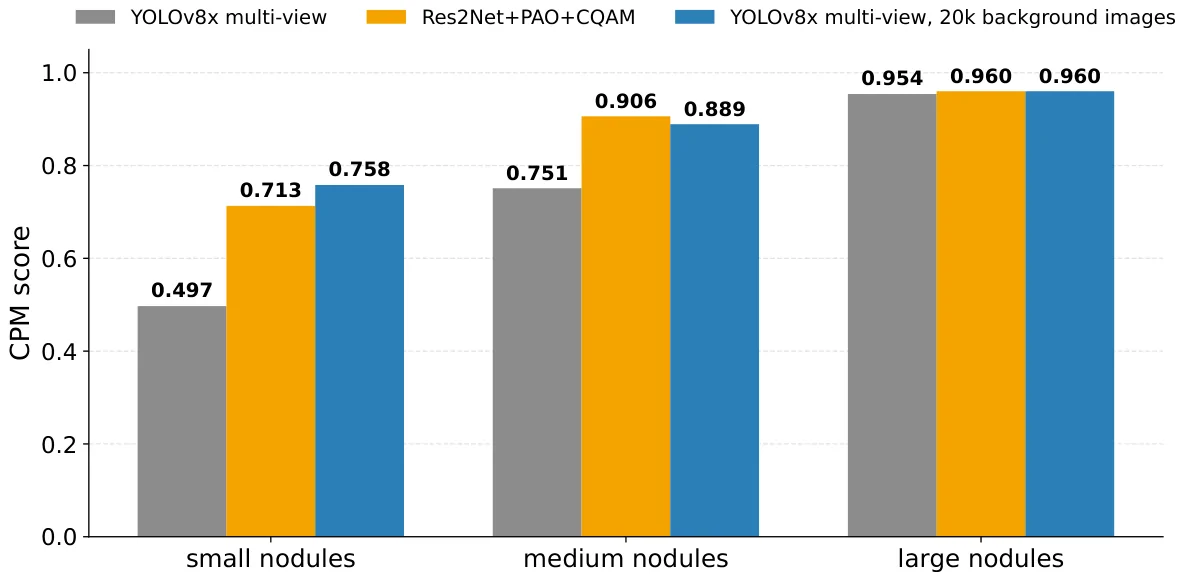
Size-stratified CPM comparison. Res2Net+PAO+CQAM values are from Zhang et al. [17]; the YOLOv8x values correspond to the experiments in this work.

Using the 20k background setting, the multi-view YOLOv8x model achieved a size-stratified CPM of 0.758 for small nodules, 0.889 for medium nodules, and 0.960 for large nodules, as shown in Figure 3. The Res2Net+PAO+CQAM values are those reported by Zhang et al. [17] under their LUNA16 evaluation setting. Because Zhang et al. report size-stratified performance as CPM values for small, medium, and large nodules, the comparison is presented at the same CPM-level granularity.

**Figure 4:**
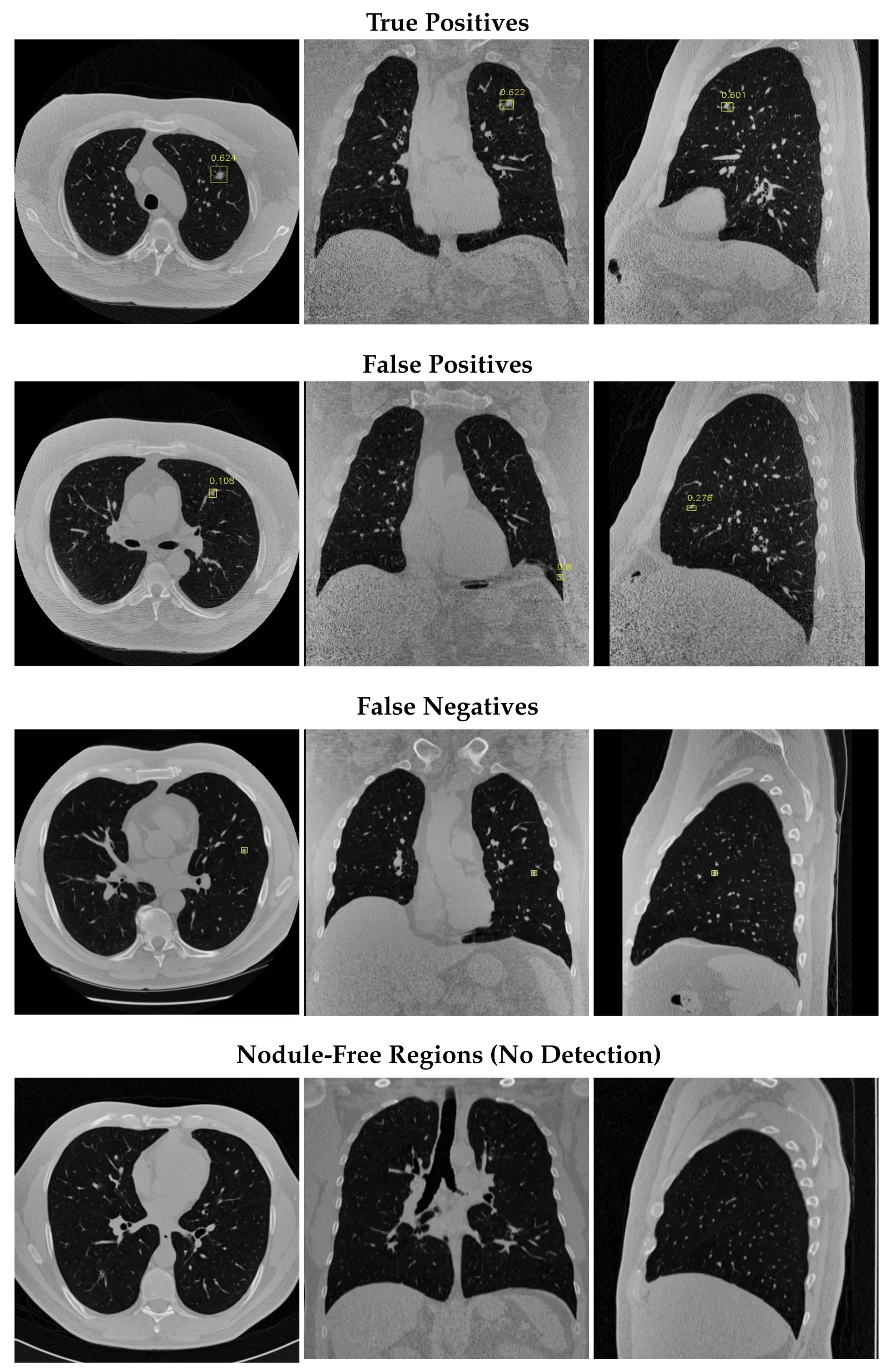
Representative qualitative results from the proposed lung-nodule detection method. The three images in each row correspond to the axial, coronal, and sagittal views, respectively. The final row shows nodule-free regions with no detection and is not used as a true-negative category in the LUNA16 FROC metric.

Figure 4 presents representative qualitative outcomes from the prediction stage. True-positive examples show official LUNA16 nodules detected by the method; false-positive examples show generated candidates that are not matched to an official nodule; and false-negative examples show official nodules that were missed. The final group illustrates nodule-free regions in which no candidate was generated.

## 6. Conclusions

This work investigated a YOLOv8x-based lung-nodule detection strategy that combines three orthogonal CT views with background-negative training and inter-view score fusion. Compared with the axial baseline, the combined strategy reduced the average candidate burden from 32.61 to 9.11 candidates per scan while increasing CPM from 0.553 to 0.863 and maintaining high sensitivity. With the three-view positive training setup held fixed, adding background samples also improved CPM and reduced the number of generated candidates.

Several limitations should be considered when interpreting these results. First, the auxiliary positive clusters were obtained from the same LIDC-IDRI CT population from which LUNA16 was derived and were included in every training fold. Therefore, strict scan-level independence was not maintained for the auxiliary set. Evaluation was restricted to the LIDC-IDRI/LUNA16 source population, and external generalizability to independent CT populations was not evaluated in this study. Second, the contribution of the auxiliary LIDC-IDRI samples was not evaluated separately. In addition, the axial-to-multi-view comparison increased the number of 2D representations per nodule, without independently quantifying the effects of view diversity and image count. Third, the 5:1:1 fusion weighting was selected based on aggregate results from the same 10-fold cross-validation protocol used for performance estimation, rather than on an independent validation set. Therefore, the reported performance of this configuration may be optimistic.

Future work should include external validation, controlled ablations of auxiliary-sample use, anatomical views, and image count, independent tuning of fusion hyperparameters, and a systematic error analysis of false-positive and false-negative patterns. We also plan to investigate fully 3D detection architectures and to test whether the background-negative strategy transfers to other detector families.

## Figures and Tables

**Table 1:** LUNA16 nodule-diameter RMSE.

Category	RMSE (mm)
small	0.7879
medium	1.8060
large	4.3246
all nodules	1.8300

**Table 2:** Distribution of auxiliary LIDC-IDRI annotation clusters with fewer than three radiologist annotations.

Nodule Size (mm)	Category	Percentage (%)	Number
<5	small	49.76	521
≥5 and <20	medium	47.37	496
≥20	large	2.87	30
			1047

**Table 3:** LUNA16 nodule distribution.

Nodule Size (mm)	Category	Percentage (%)	Number
< 5	small	22.76	270
≥ 5 and < 20	medium	73.02	866
≥ 20	large	4.22	50
			1186

**Table 4:** Combined distribution of official LUNA16 nodules and auxiliary LIDC-IDRI annotation clusters.

Nodule Size (mm)	Category	Percentage (%)	Number
<5	small	35.42	791
≥5 and <20	medium	60.99	1362
≥20	large	3.58	80
			2233

**Table 5:** Comparison of CPM, sensitivity, and candidate burden for different background budgets. All experiments use YOLOv8x.

Training Views (Nodules)	Training Views (Background)	Prediction Views	Avg. Cand/Scan	Sensitivity	CPM
ax	ax–0k	ax	32.61	0.908	0.553
ax, co, sg	ax–0k	ax, co, sg	31.09	0.933	0.702
ax, co, sg	ax–5k	ax, co, sg	17.10	0.958	0.832
ax, co, sg	ax–10k	ax, co, sg	12.00	0.953	0.844
ax, co, sg	ax–15k	ax, co, sg	9.83	0.936	0.847
ax, co, sg	ax–20k	ax, co, sg	9.11	0.937	0.863

**Table 6:** Aggregate CPM for the tested axial:coronal:sagittal fusion weights using YOLOv8x with the 20k background configuration.

Training Views (Nodules)	Training Views (Background)	Prediction Views	Fusion Weights (ax, co, sg)	CPM
ax, co, sg	ax–20k	ax, co, sg	2, 1, 1	0.846
ax, co, sg	ax–20k	ax, co, sg	3, 1, 1	0.854
ax, co, sg	ax–20k	ax, co, sg	4, 1, 1	0.857
ax, co, sg	ax–20k	ax, co, sg	5, 1, 1	0.863
ax, co, sg	ax–20k	ax, co, sg	6, 1, 1	0.863

**Table 7:** Comparison with published LUNA16 results. Sensitivity is reported at the seven standard FROC operating points. For our method, the values correspond to the bootstrap-mean FROC sensitivities produced by the LUNA16 evaluation procedure with 1000 scan-level resamples.

Methods	Average Number of FPs/Scan							CPM
	**0.125**	**0.25**	**0.5**	**1**	**2**	**4**	**8**	
Xie et al. [16] ^a^	0.439	0.688	0.796	0.852	0.864	0.864	0.864	0.767
Gu et al. [21]	0.480	0.649	0.792	0.879	0.916	0.929	0.930	0.797
Pezeshk et al. [22]	0.637	0.723	0.804	0.865	0.907	0.938	0.952	0.832
Dou et al. [14]	0.659	0.745	0.819	0.865	0.906	0.933	0.946	0.839
Zhu et al. [15]	0.692	0.769	0.824	0.865	0.893	0.917	0.933	0.842
Zhang et al. [17]	0.732	0.774	0.830	0.866	0.917	0.929	0.946	0.856
Ours	0.712	0.788	0.852	0.892	0.921	0.936	0.937	0.863
Jian et al. [19]	0.764	0.821	0.857	0.892	0.928	0.942	0.957	0.880
Liu et al. [18]	0.865	0.887	0.901	0.916	0.927	0.932	0.958	0.912

^a^ The Xie et al. [16] values are reproduced from Table 3 of Zhang et al. [17]. That source reports a CPM of 0.775; the arithmetic mean of its seven listed sensitivities is 0.7667.

## Data Availability

The LIDC-IDRI dataset analyzed in this study is publicly available from The Cancer Imaging Archive (TCIA) at https://www.cancerimagingarchive.net/collection/lidc-idri/ (accessed on 23 September 2026). The LUNA16 dataset is publicly available at https://luna16.grand-challenge.org/ (accessed on 23 September 2026). The experimental results supporting the findings of this study are reported in the manuscript. No additional derived datasets are available.

## Abbreviations

The following abbreviations are used in this manuscript:

2D	Two-Dimensional
3D	Three-Dimensional
CAD	Computer-Aided Detection
CNN	Convolutional Neural Network
CNPq	National Council for Scientific and Technological Development
CPM	Competition Performance Metric
CT	Computed Tomography
FN	False Negative
FP	False Positive
FROC	Free-Response Receiver Operating Characteristic
GPU	Graphics Processing Unit
LIDC-IDRI	Lung Image Database Consortium and Image Database Resource Initiative
LUNA16	LUng Nodule Analysis 2016
RMSE	Root Mean Square Error
TCIA	The Cancer Imaging Archive
TP	True Positive
YOLO	You Only Look Once
